# Volatile organic compounds of *Hanseniaspora uvarum* increase strawberry fruit flavor and defense during cold storage

**DOI:** 10.1002/fsn3.1116

**Published:** 2019-07-06

**Authors:** Luyao Wang, Guoxia Dou, Hongna Guo, Qiuqing Zhang, Xiaojie Qin, Wei Yu, Chunhao Jiang, Hongmei Xiao

**Affiliations:** ^1^ Key Laboratory of Quality and Safety Risk Assessment in Agricultural Products Preservation (Nanjing), Ministry of Agriculture, College of Food Science and Technology Nanjing Agricultural University Nanjing China; ^2^ Department of Plant Pathology, College of Plant Protection Nanjing Agricultural University Nanjing China; ^3^ State Key Lab of Crop Genetics and Germplasm Enhancement, Cytogenetics Institute Nanjing Agricultural University Nanjing China; ^4^ MOST‐USDA Joint Research Center for Food Safety and Bor Luh Food Safety Center, School of Agriculture and Biology Shanghai Jiao Tong University Shanghai China

**Keywords:** defense‐related enzymes, GC‐MS, *Hanseniaspora uvarum*, strawberry, volatile organic compounds

## Abstract

Volatile organic compounds (VOCs) of antagonistic yeasts are considered as environmental safe fumigants to promote the resistance and quality of strawberry (*Fragari*a *ananassa*). By GC‐MS assays, VOCs of *Hanseniaspora uvarum* (*H. uvarum*) fumigated strawberry fruit showed increased contents of methyl caproate (5.8%), methyl octanoate (5.1%), and methyl caprylate (10.9%) in postharvest cold storage. Possible mechanisms of *H. uvarum* VOCs involved in regulations of the defense‐related enzymes and substances in strawberry were investigated during postharvest storage in low temperature and high humidity (2 ± 1°C, RH 90%–95%). Defense‐related enzymes assays indicated *H. uvarum* VOCs stimulated the accumulation of CAT, SOD, POD, APX, PPO, and PAL and inhibited biosynthesis of MDA in strawberry fruit under storage condition. Moreover, the expression levels of related key enzyme genes, such as *CAT*, *SOD*, *APX42*, *PPO*, and *PAL6*, were consistently increased in strawberry fruit after *H. uvarum* VOCs fumigation.

## 1 INTRODUCTION

Fungal infections bring serious loss in strawberry production, especially during field and postharvest phases (Paulus, 1990). Many studies reported successful preventing of postharvest decay with chemical fungicides (El‐Mougy, El‐Gamal, & Abdalla, 2008; Sallato, Torres, Zoffoli, & Latorre, 2007). To date, there is much concern about fungicide residue in agricultural product, and biocontrol has been considered as a more acceptable method for controlling postharvest diseases (Karunaratne, 2011; Kilani‐Feki et al., 2016; Mohamed & Saad, 2009; Pretorius, Van, & Clarke, 2015). Multiple biocontrol agents (BCAs) have been isolated from environment, and they were shown to facilitate the control of postharvest decay of strawberry fruit (El Ghaouth & Wilson, 2002; Menel, Faten, & Moktar, 2012; Wei, Mao, & Tu, 2014; Zhang et al., 2007), suggesting possible application of BCAs in strawberry postharvest storage. Some yeast strains with potential biocontrol ability directly inhibit the growth of fungal pathogens on strawberry by antagonism pattern (Wei et al., 2014; Zhang et al., 2007). Different mechanisms were involved in antagonistic microorganism‐mediated fungal pathogen growth inhibition, and volatile organic compounds (VOCs) have been suggested to act as a functional molecular factor to interfere fungal pathogen growth (Asari, Matzén, Petersen, Bejai, & Meijer, 2016; Sánchez‐Fernández et al., 2016). It was also reported that VOCs treatment may control postharvest fungal disease and increase the storage period of strawberry fruit (Archbold, Hamiltonkemp, Barth, & Langlois, 1997).

Plant resistance could be triggered by various biotic and abiotic stresses and was known as induced system resistance (ISR; Choudhary & Johri, 2009; Heil & Bostock, 2002). Plenty of studies indicated BCAs decreasing the fruit postharvest decay by enhancing defense‐related enzyme activities. A study on peach showed antagonistic yeast strains, including *P. membranifaciens*, *C. laurentii*, *Candida guilliermondii*, and *Rhodotorula glutinis*, can stimulate activities of catalase (CAT), peroxidase (POD), and superoxide dismutase (SOD; Xu, Qin, & Tian, 2008). In another study on apple fruit, it showed Aureobasidium pullulans induced the activities of chitinase, glucanase, and peroxidase in fruit tissue after 24 hr (Ippolito, Elghaouth, Wilson, & Wisniewski, 2000).

One of those antagonistic yeast strain that inhibits the growth of *B. cinerea* on strawberry fruit is *H. uvarumi*, probably by nutrients and space competition, host defense induction, morphology change of hyphae, and synthesis of secondary metabolites (Cai, Yang, Xiao, Qin, & Si, 2015; Qin et al., 2013). Preharvest application of *H. uvarum* on strawberry fruit was able to stimulate the enzymes associated with active oxygen metabolism, such as POD, polyphenol oxidase (PPO), SOD, phenylalanine ammonialyase (PAL), and CAT (Cai et al., 2015).

Although it is relatively efficient to control postharvest diseases by applying biocontrol yeast strains directly on strawberry fruit, BCAs treatment might increase the risk of microbe contamination during marketing stage. Thus, VOCs treatment has a broader prospect instead of directly spraying BCAs culture on strawberry fruit surface in postharvest stage. However, little is known about these specific mechanisms of controlling strawberry postharvest diseases of by VOCs.

In previous study, VOCs produced by *H. uvarum* showed effective inhibition to the growth of *B. cinerea* in vitro (Cai et al., 2015). Here, we explore the further functions of VOCs produced by *H. uvarum* by testing possible alterations in strawberry volatile emissions, as well as the variations of defense‐related enzymes and substances in strawberry fruit during postharvest storage period.

## 2 MATERIALS AND METHODS

### 2.1 *H. uvarum* strain and culture

*Hanseniaspora uvarum* (Genbank accession number: JX125041) was isolated from the surface of strawberry and maintained in PDA medium (200 g/L potato, 20 g/L dextrose, 15 g/L agar).

*Hanseniaspora uvarum* strain was grown in PDB liquid medium (200 g/L potato, 20 g/L dextrose) at 28°C for 24 hr in a gyratory shaker at 200 g. The strain culture was centrifuged at 6,000 *g* for 15 min at 4°C, and the yeast strain precipitate was then adjusted to final concentration of 1 × 10^9 ^CFU/ml with sterile distilled water with 0.05% Tween‐20. Five hundred microliter of yeast suspension was inoculated on the PDA dishes for the preparation of VOCs treatment.

### 2.2 Fruit and VOCs treatment

Fruit of* Fragaria ananassa* “Hong Yan” was harvested in early morning from a farm in Yuhua district, Nanjing city, Jiangsu province, China. The strawberry fruit was then transported to laboratory condition immediately within 2 hr. A total of 300 strawberry fruits in similar shape and size and without physical injuries or microorganisms infection were randomly selected.

*Hanseniaspora uvarum* VOCs treatment on strawberry fruit was conducted in sealed glass desiccators (9 cm by 27 cm, down diameter by up diameter, with a hollow clapboard inside) as previously described (Qin, Xiao, Cheng, Zhou, & Si, 2017). Eight uncovered Petri dishes with *H. uvarum* culture on PDA medium were placed on the bottom of the glass desiccator (use noninoculated PDA medium as negative control), and fresh strawberries were placed on the hollow clapboard. All desiccators were sealed by parafilm and incubated at 2°C for 3 days. Then, all treated strawberry fruits were placed in normal storage condition (2 ± 1°C, RH 90%–95%) with appropriate aeration system. Strawberry fruit samples were collected for further biochemical assessment after 0, 3, 8, 13, 18, and 25 days, respectively.

### 2.3 Detection of defense‐related enzymes in strawberry fruit

The enzyme activities of strawberry fruit were measured following the methods described in previous experiment with some modifications (Cai et al., 2015). Strawberry fruit samples (2.0 g for each treatment) were homogenized in 8 ml ice‐cold phosphate buffer (50 mmol/L, pH 7.8, containing 1% polyvinylpyrrolidone [PVP]) and then centrifuged at 12,000 *g* for 20 min at 4°C, and supernatant was collected as crude extract. Each strawberry fruit treatment had three parallel lines, and the test was repeated twice.

For POD activity measurement, reaction buffer (1 ml 0.1 mol/L acetic acid buffer, 1 ml crude extract, 1 ml 2‐methoxyphenol, 0.4 ml 0.75% H_2_O_2_) was mixed with crude extract of strawberry fruit. Each variation (0.001) of the mixture absorbance at 460 nm within 2 min represents 1 unit of the enzyme activity. The specific activity was expressed as units per gram of fresh weight.

The SOD activity of strawberry fruit was evaluated by following procedures. The reaction buffer (2.0 ml 50 mmol/L, pH 7.8 phosphate buffer, 0.2 ml 30 mmol/L L‐methionine, 0.2 ml 750 mol/L nitrotetrazolium blue chloride, 0.3 ml 20 µmol/L riboflavin) was gently mixed with 0.5 ml crude extract, and reaction mixture was then exposed by fluorescent lamp (400 lx) for 10 min. Each enzyme unit of SOD activity was defined as 50% inhibition rate of the photochemical reaction displayed by absorbance at 560 nm, and the reaction mixture without exposure treatment was considered as blank. The specific activity was expressed as units per gram of fresh weight.

For CAT activity measurement, reaction buffer (3 ml sodium phosphate buffer, 0.4 ml 0.75% H_2_O_2_) was mixed with 0.2 ml crude extract. Each variation (0.001) of the mixture absorbance at 240 nm within 2 min represents 1 unit of the CAT enzyme activity. The specific activity was expressed as units per gram of fresh weight.

For APX activity measurement, reaction buffer (3 ml sodium phosphate buffer, 0.2 ml 0.01 mol/L L‐ascorbic acid, 0.2 ml 0.75% H_2_O_2_) was gently mixed with 0.2 ml crude extract. Each variation (0.001) of the mixture absorbance at 290 nm within 2 min represents 1 unit of the APX enzyme activity. The specific activity was expressed as units per gram of fresh weight.

For activity of PAL and PPO, content measurement of MDA, and superoxide anion in strawberry fruit, the procedures were as described in previous study (Cai et al., 2015).

### 2.4 Volatile compound analysis on strawberry fruit

Top parts of three strawberry fruits for each treatment were collected and placed in headspace sampling tubes. GC‐MS analysis was then conducted as following steps. A 2‐cm fused‐silica fiber coated with divinylbenzene/carboxen/polydimethylsiloxane 50/20 μm (DBV/CAR/PDMS) was inserted into the side of the sampling tubes via a silicone septum after 15 min of headspace equilibration. Volatile compounds were collected from headspace sampling tubes for 30 min and then desorbed into GC injector for 5 min at 250°C. Separation was achieved on an HP‐Innowas fused‐silica capillary column. The GC oven temperature program consisted of 40°C for 2.5 min, raised from 40 to 200 at 5°C/min, and then to 240°C for 5 min at 10°C/min. Helium was used as carrier gas with a constant column flow rate of 0.004 mol/hr. Compounds exiting the column were ionized via electron impact at 70 eV and scanned with a quadrupole mass spectrometer with a m/z range between 30 and 300 Th. The volatile profile of each sample was reported as absolute peak area.

### 2.5 Real‐time PCR analysis of mRNA abundance

Fresh strawberry fruit for defense relevant gene test was treated with *H. uvarum* VOCs for 1, 2, and 3 days, and then, strawberry samples (0.1 g for each) were collected and ground into powder with liquid N_2_. All the microbe treatments followed the method described in fruit and VOCs treatment section. Total RNA was extracted using 1 ml TRIZOL reagent (Invitrogen Co.) and was treated with RNase‐free DNAse (Takara Co.) to remove genomic DNA. Reverse transcription of RNA was performed with 1 μg of total RNA using M‐MLV reverse transcriptase, according to the manufacturer's protocol (Invitrogen Co.). The primers used were listed in Table 1. A constitutively expressed gene (18S rRNA in *Fragaria x ananassa*) was a reference gene in quantitative real‐time PCR analysis. Three replicates were performed for each treatment.

**Table 1:** Sequence of the primers used for genes expression in strawberry

Gene	Genebank	Forward primer sequence (5′–3′)	Reverse primer sequence (5′–3′)
*PAL6*	HM641823.1	GTGAAAGAAGCGAAGAAGG	GAAGCTCGGAGCAGTATG
*CAT*	KC433883.1	GTCTTCTTCGTCCGTGAT	GTAGTTCCCAGCAGCAAT
*PPO*	EU523113.1	GGAGGCGGAGCAATAGAGAC	AGCCAGACCCCCTCAATACT
*APX42*	AF159630.1	CACAAGGAACGGTCTGGATT	CGCAGCGTATTTCTCAACAA
*SOD*	–	ATGGTGCTCCTGAAGATGA	TAGAGTGTGGTCCAGTGAG
18S rRNA	X15590.1	TGTGAAACTGCGAATGGCTCATTAA	GAAGTCGGGATTTGTTGCACGTATT

### 2.6 Statistical analysis

All statistical analyses were performed in the SAS Software (version 8.2; SAS Institute). Comparison of means was performed by Duncan's multiple range tests. Statistical significance was assessed at the level of *p < *0.05.

## 3 RESULTS

### 3.1 Effects of *H. uvarum* VOCs on gray mold decay of strawberry during cold storage

**Figure 1:**
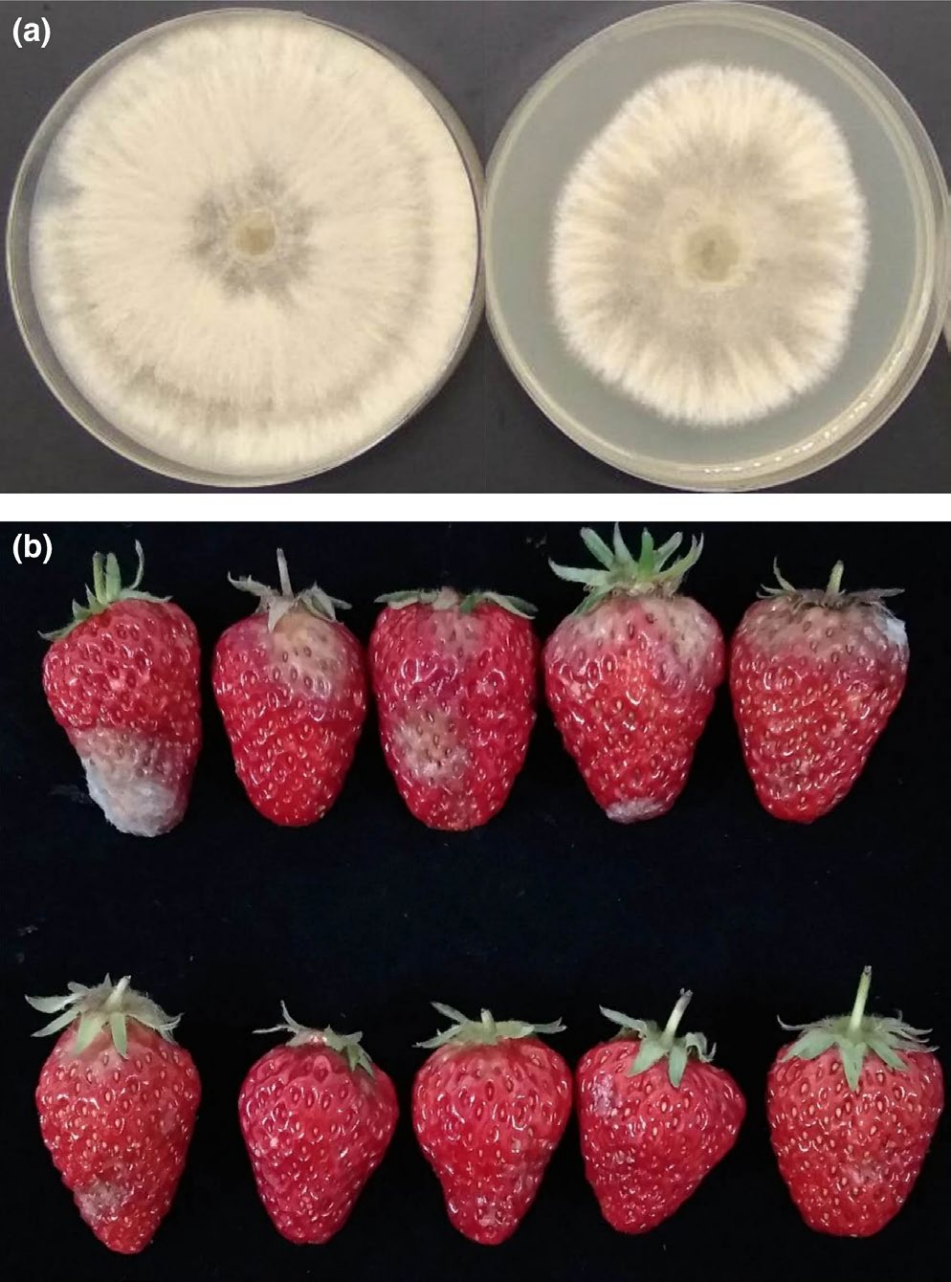
Effects of *H. uvarum* volatile organic compounds (VOCs) to *B. cinera* and strawberry fruit. (a) *H. uvarum* VOCs obviously inhibit in vitro growth of *B. cinera* in 7 days. (b) Changes in appearance of *H. uvarum* VOC‐treated strawberry fruit (down) and control group (up) after cold storage for 25 days. Three biological replicates were performed for each assay with similar results

*Hanseniaspora uvarum* is an effective BCA in preventing postharvest diseases (Cai et al., 2015), and its VOCs also showed positive effects in prolonging shelf life of strawberry fruit (Qin et al., 2017). Further experiments were conducted to understand the functions of *H. uvarum* VOCs in controlling strawberry postharvest diseases. Gray mold decay occurs during low‐temperature storage of strawberry fruit, usually caused by physical damage and infections of fungal pathogen spores, such as *Botrytis cinera* on fruit surface. In Figure 1a, *H. uvarum* VOCs inhibited the in vitro growth of *B. cinerea* on PDA plate in 7 days (Figure 1a, right), while the hyphae of *B. cinerea* had spread on whole plate within 3 days without treatment (Figure 1a, left). Figure 1b, which showed the appearance of *H. uvarum* VOCs treated or nontreated strawberry fruit at 25 dpt (day post‐treatment), suggests the decay severity of *H. uvarum* VOC‐treated strawberry fruits was obviously lower than that of nontreated fruit, and *H. uvarum* VOCs was able to restrain the spontaneous happening of gray mold decay symptom on strawberry fruit in cold storage condition. These results obviously showed that the *H. uvarum* VOCs treatment can maintain the quality of strawberry during cold storage period.

### 3.2 *H. uvarum* VOCs altered content of organic esters in strawberry volatile emissions

**Figure 2:**
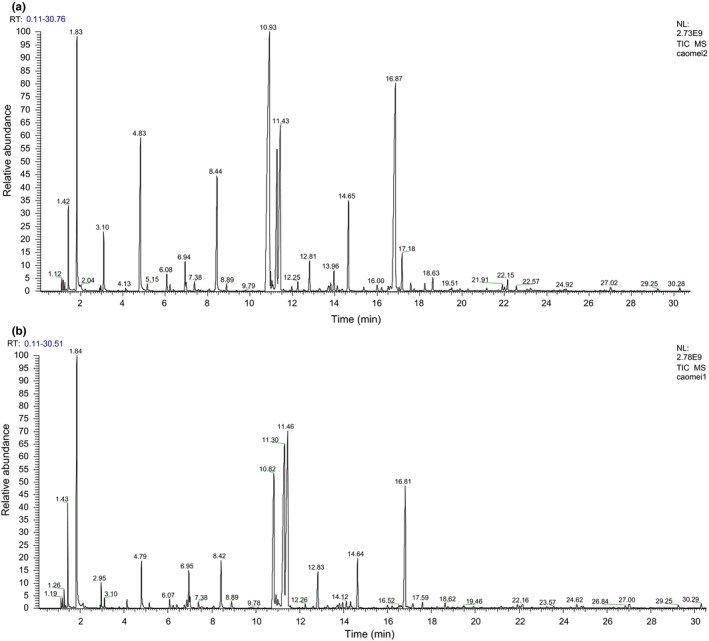
GC‐MS analysis on strawberry volatile emissions 5 days after *H. uvarum* volatile organic compounds (VOCs) (a) and mock treatments (b). Three biological replicates were performed for each assay with similar results

The contents of organic esters are main flavor factors of strawberry fruit, which derived from multiple esterification reactions during strawberry postharvest storage. According to the results of GC‐MS analysis, both control group and *H. uvarum* VOCs treatment showed increased contents of organic esters in strawberry fruit VOCs, and the increments of several specific organic esters were higher in *H. uvarum* VOCs treatment (Figure 2). Table 2 lists all detected strawberry volatile emissions of strawberry fruit after 5 days of room temperature storage. By *H. uvarum* VOCs treatment, the strawberry fruit released 5.8% more of methyl acetate, 5.1% more of methyl caprylate, and 10.9% more of ethyl octanoate after 5 days compared with strawberry fruit before storage (Table 2). In addition, nontreated strawberry fruit showed slightly lower increments of methyl acetate, methyl caprylate, and ethyl octanoate (3.7%, 4.4%, and 8.9%, respectively, in Table 2). These results indicated that *H. uvarum* VOCs treatment might improve the flavor of strawberry fruit by increasing the contents of specific esters during postharvest storage.

**Table 2:** Strawberry volatile organic compounds tested 5 days after *H. uvarum* VOCs treatment and their peak area percentages

RT (retention time)	Compound name	Molecular formula	Peak area percentage		
			CAS (registration number)	CK	*H. uvarum*
1.12	Nitrogen oxide	N_2_O	20621‐02‐7	0.18	0.64
1.19	2‐aminoethyl alcohol	C_2_H_7_NO	141‐43‐5	0.89	0.39
1.27	Ethyl alcohol	C_2_H_6_O	64‐17‐5	1.2	0.39
1.42	Methyl n‐acetylglycine	C_5_H_9_NO_3_	1117‐77‐7	7.6	4.1
1.84	Ethyl acetate	C_4_H_8_O_2_	141‐78‐6	17.7	12.9
2.90	Ethyl propionate	C_5_H_10_O_2_	105‐37‐3	1.8	0.26
3.10	Methyl butyrate	C_5_H10O_2_	623‐42‐7	0.7	3.2
3.78	Ethyl isobutyrate	C_6_H_12_O_2_	97‐62‐1	0.7	0.13
3.97	Ethyl butyrate	C_6_H_12_O_2_	105‐54‐4	0.18	0.13
4.13	Isobutyl acetate	C_6_H_12_O_2_	110‐19‐0	0.18	0.26
4.19	Methyl 2‐methylbutyrate	C_6_H_12_O_2_	868‐57‐5	–	0.39
4.79/4.82	Ethyl butyrate	C_6_H_12_O_2_	105‐54‐4	3.5	0.13
5.15	Butyl acetate	C_6_H_12_O_2_	123‐86‐4	–	0.13
5.87	Propyl butyrate	C_11_H_14_O_2_	105‐66‐8	–	0.26
6.07	Ethyl 2‐methylbutyrate	C_7_H_14_O_2_	7452‐79‐1	0.7	0.77
6.23	Ethyl isovalerate	C_7_H_14_O_2_	108‐64‐5	0.36	0.39
6.95	Isoamyl acetate	C7H14O2	123‐92‐2	2.7	1.3
7.38	Isoamyl acetate	C_7_H_14_O_2_	123‐92‐2	0.53	0.64
8.41/8.44	Methyl hexanoate	C_7_H_14_O_2_	106‐70‐7	3.7	5.8
8.76	Methyl hex‐3‐enoate	C_7_H_12_O_2_	2396‐78‐3	0.36	0.13
8.89	Ethyl tiglate	C_7_H_14_O_2_	5837‐78‐5	0.53	0.39
9.78	Methyl ester	C_7_H_12_O_2_	13894‐63‐8	0.18	0.26
10.10	2‐Pentanol	C_8_H_16_O_2_	108‐84‐9	0.18	7.1
10.80/10.82	Ethyl caproate	C_8_H_16_O_2_	123‐66‐0	9.8	12.9
11.08	(E)‐2‐Hexen‐1‐ol	C_8_H_14_O_2_	2497‐18‐9	1.8	3.9
11.27/11.29	2‐Pentanol	C_8_H_16_O_2_	108‐84‐9	11.5	6.4
11.42/11.45	Octyl acetate	C_10_H_20_O_2_	112‐14‐1	12.8	8.3
12.82	Methoxy‐2,5‐dimethyl‐3(2H)‐furanone	C_6_H_8_O_2_	4077‐47‐8	3.5	1.9
13.72	Propyl hexanoate	C_9_H_18_O_2_	626‐77‐7	0.53	–
14.65	Methyl octanoate	C_9_H_18_O_2_	111‐11‐5	4.4	5.1
16.81/16.86	Ethyl caprylate	C_10_H_20_O_2_	106‐32‐1	8.9	10.9
17.59	Butyric anhydride	C8H14O3	106‐31‐0	0.53	–
18.62	phenethyl acetate	C_10_H_12_O_2_	103‐45‐7	0.18	0.64
19.20	1‐nonanecarboxylic acid	C_10_H_20_O_2_		0.18	–
19.46	Tridecane	C_13_H_28_	629‐50‐5	0.18	0.13
19.51	Ethyl nonanoate	C_11_H_22_O_2_	123‐29‐5	0.36	0.26
19.92	Nonyl acetate	C_11_H_22_O_2_	143‐13‐5	0.18	0.13
22.15	Ethyl caprate	C_12_H_24_O_2_	110‐38‐3	0.36	0.64
24.92	2,4‐bis(1,1‐dimethylethyl)‐6‐methyl‐	C_15_H_24_O	616‐55‐7	0.18	0.26
27.01	1,1,1‐trimethyl‐,1,1′,1″‐triester with boric acid (H3BO3)	C_9_H_28_Si_4_	4325‐85‐3	0.18	0.26
29.25	Heptadecane	C_17_H_36_	629‐78‐7	0.18	0.26

### 3.3 Effects of *H. uvarum* VOCs on defense‐related enzymes and substances of strawberry during cold storage

Microbe VOCs were reported for inducing the synthesis of plant defense‐related enzymes during biocontrol process in many reports (Giorgio, Stradis, Cantore, & Iacobellis, 2015; Raza, Ning, Yang, Huang, & Shen, 2016). To explore the role of *H. uvarum* VOCs further, we measured the contents of different defense‐related enzymes and substances on strawberry fruit treated by *H. uvarum* VOCs in cold storage period.

**Figure 3:**
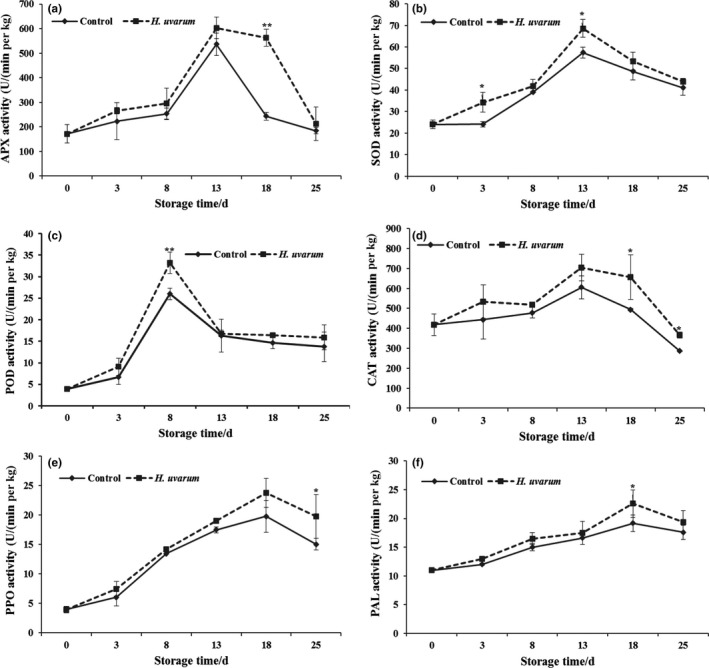
Effect of fumigation with *H. uvarum* volatile organic compounds (VOCs) on defense‐related enzymes in strawberry fruit during cold storage. (a) APX, (b) SOD, (c) POD, (d) CAT, (e) PPO, (f) PAL. Each value is the mean for three independent replicates. Asterisks indicate statistical differences compared to control according to Duncan's multiple range test at *p* ≦ 0.05 level. The vertical bar indicates the standard error. Three biological replicates were performed for each assay with similar results

In assays of APX activity in strawberry fruit, the APX enzyme activities of *H. uvarum* VOC‐treated fruit were slightly higher than nontreatment group on the 3, 8, 13, and 25 dpt. The peak value of APX enzyme activity was observed on day 13 in *H. uvarum* VOC‐treated fruit which is approximately 600 U/(min g kg^−1^), and the APX activity of treated fruit was significantly higher (*p* < 0.05) than that of the nontreatment group on 18 dpt (Figure 3a). As shown in Figure 3b, under the cold storage condition, SOD accumulation peaked at 13 dpt in strawberry fruit of both *H. uvarum* VOCs treatment and nontreatment groups, and the SOD enzyme activity of *H. uvarum* VOC‐treated fruit was significantly higher (*p* < 0.05) than nontreatment group at 3 and 13 dpt. In the strawberry fruit during storage period, POD activity reached a maximum at 8 dpt in both *H. uvarum* VOCs treatment and nontreatment groups, and the POD activity of treatment group was induced approximately 1.2‐fold than that of the nontreatment group (Figure 3c). In Figure 3d, it was shown that the CAT activity of *H. uvarum* VOC‐treated fruit was significantly higher (*p* < 0.05) than that of the control group from 3 to 25 dpt, and the CAT activity in *H. uvarum* VOCs treatment group has been increased by 16.18% compared with nontreatment group at the end of storage.

Under the condition of *H. uvarum* VOCs treatment, the PPO activity in strawberry fruits was slightly higher than that in nontreatment group from 0 to 13 dpt (Figure 3e). The PPO activity reached the peak at 18 dpt in both *H. uvarum* VOCs treatment and nontreatment groups and was approximately 1.2‐fold in treated strawberry fruit compared with nontreated strawberry fruit. The PPO activity of *H. uvarum* VOCs treatment group was significantly (*p* < 0.05) higher than that of nontreatment group at the end of storage period (18 and 25 dpt; Figure 3e). In consistent with PPO activity, the results of PAL activity assay displayed similar tendency. The peak values of PPO activity in both *H. uvarum* VOCs treatment and nontreatment groups were observed at 18 dpt, and the PAL activity of treatment group was significantly (*p* < 0.05) higher than that of control group at the 18 dpt in cold storage condition (Figure 3f).

**Figure 4:**
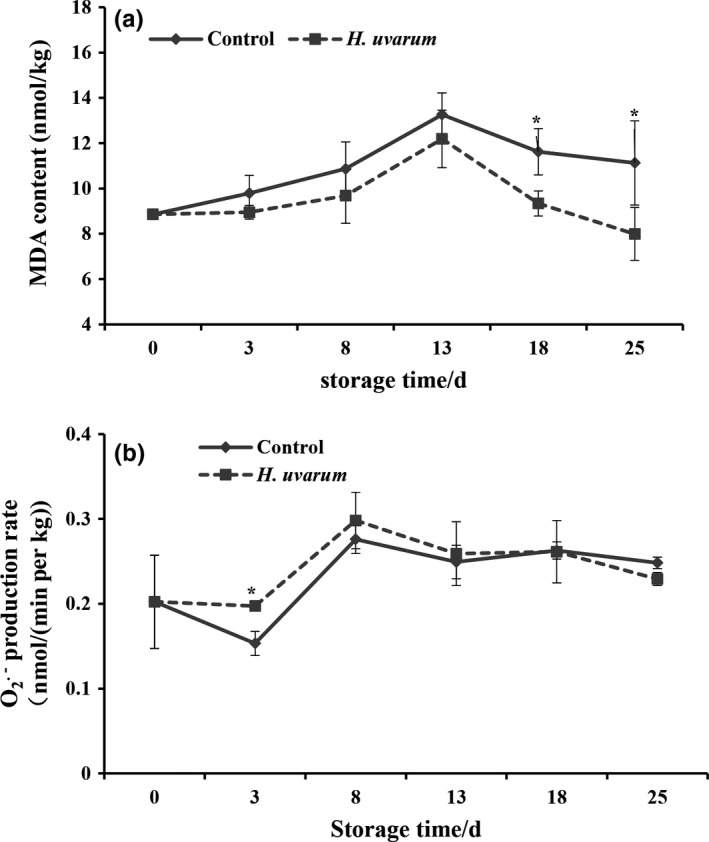
Effect of fumigation with *H. uvarum* volatile organic compounds (VOCs) on MAD content (a) and O^2−^ production rate (b) in strawberry fruit during cold storage. Each value is the mean for three independent replicates. Asterisks indicate statistical differences compared to control according to Duncan's multiple range test at *p* ≦ 0.05 level. The vertical bar indicates the standard error, and three biological replicates were performed for each assay with similar results

Next, we examined whether the *H. uvarum* VOCs treatment is able to trigger the change of MDA and superoxide anion production contents in strawberry fruit during storage period. Figure 4a showed MDA contents change in strawberry fruit during cold storage condition up to 25 dpt. The peak values of MDA contents were observed at 13 dpt in both *H. uvarum* VOCs treatment group and nontreatment group. The MDA content accumulation in *H. uvarum* VOCs treatment group was significantly lower (*p* < 0.05) than that of the nontreatment group at 18 and 25 dpt. In Figure 4b, the superoxide anion production was significantly increased in *H. uvarum* VOC‐treated strawberry fruit in 3 dpt (*p* < 0.01) and 8 dpt (*p* < 0.05). After 13 dpt, the superoxide anion production in treated strawberry fruit kept in the same level with nontreated fruit (Figure 4b) indicates the influence of *H. uvarum* VOCs on superoxide anion production in strawberry fruit is more like in early stage of postharvest storage.

### 3.4 Effects of *H. uvarum* VOCs on expression levels of defense‐related enzyme genes in strawberry

**Figure 5:**
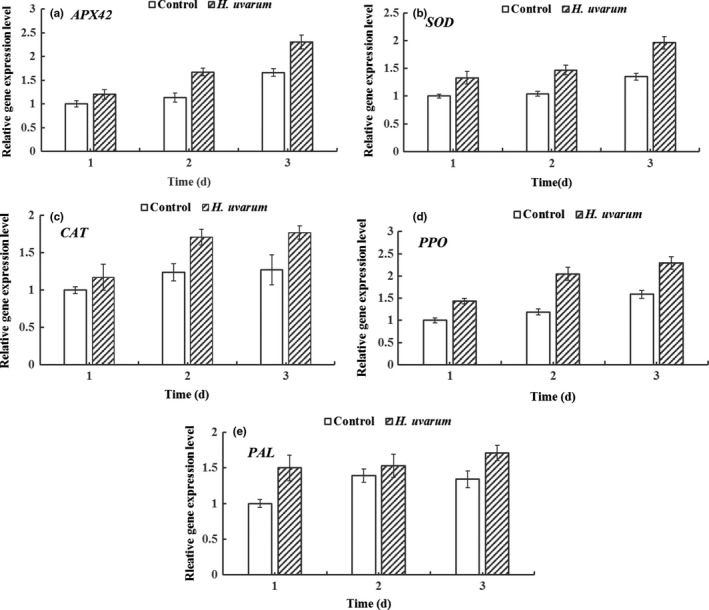
Effect of fumigation with *H. uvarum* volatile organic compounds (VOCs) on expression levels of defense‐related enzyme genes. Expression levels of *SOD* (a), *CAT* (b), *PAL* (c), *PPO* (d), and *APX42* (e) were detected in strawberry fruit during cold storage. Each value is the mean for three independent replicates. Asterisks indicate statistical differences compared to control according to Duncan's multiple range test at *p* ≦ 0.05 level. The vertical bar indicates the standard error, and three biological replicates were performed for each assay with similar results

Expression levels of defense‐related enzyme genes were analyzed in strawberry fruit on 1, 2, and 3 days during *H. uvarum* VOCs fumigation. In this study, relative expression levels of key enzyme genes involved in biosynthesis of APX, SOD, CAT, PPO, and PAL in strawberry fruit were shown in Figure 5. Basically, expression levels of *SOD*, *PPO*, and *PAL* were significantly induced after 1 day of *H. uvarum* VOCs treatment in Figure 5b,d,e. Expression levels of *APX*, *SOD*, *CAT*, and *PPO* were significantly induced after 2 days of *H. uvarum* VOCs treatment in Figure 5a–e. All tested genes, including *APX*, *SOD*, *CAT*, *PPO*, and *PAL*, were significantly induced after 3 days of *H. uvarum* VOCs treatment in Figure 5a–e.

## 4 DISCUSSION

The proposed involvement of the *H. uvarum* volatile production in maintaining quality of strawberry fruit in cold storage period is consistent with its known roles in controlling gray mold caused by *B. cinerea* by directly applying *H. uvarum* cells on fruit (Cai et al., 2015; Qin et al., 2017).

Since strawberry is one of the popular fruits with high economic value, studies of reducing postharvest loss and maintaining fruit quality of strawberry attract wide attentions. Previous studies demonstrated some functions of *H. uvarum* in controlling the postharvest diseases of strawberry, such as inhibiting of *B. cineare* infection. Possible explanations are as follows: *H. uvarum* was able to compete nutrient and reproduction space with other microbes on strawberry surface, and some specific chemicals produced by *H. uvarum* cells could suppress conidial germination and hyphal growth of *B. cinerea* (Cai et al., 2015). Thus, it was important to examine the effects of possible productions of *H. uvarum* on strawberry fruit during storage condition. A study of Qin et al. (2017) indicated that *H. uvarum* VOCs directly inhibit in vitro fungal growth and prolong the shelf life of strawberry fruit. Yet, they have not investigated further effects and mechanisms of *H. uvarum* VOCs.

One of the aims of this study is to demonstrate whether *H. uvarum* VOCs treatment could alter specific contents in strawberry volatile emissions. Our results showed that varieties of organic esters were produced in strawberry during postharvest storage, which makes most of the contributions to the strawberry's flavor. Our previous data indicated that volatile emissions of “Hong Yan” mostly were methyl caproate, methyl butyrate, ethyl butyrate, and butyl acetate, which is similar with presented results in Table 2. It is interesting to see that *H. uvarum* VOCs treatment could increase some of the specific organic esters in strawberry fruit in postharvest storage, which might help improve the flavor and commercial value.

Furthermore, APX is known as a key enzyme in the ascorbic acid–glutathione cycle reaction, which can remove H_2_O_2 _in plants; SOD is one of the important active oxygen scavenging enzymes in plants and delays the aging process of fruit; CAT is one of the main removal enzymes of H_2_O_2_ in plants and reduces the toxic effects of H_2_O_2_ and other free radicals on plant cells; POD has the capability to remove H_2_O_2 _from plants and delays fruit senescence; and PAL is responsible for the biosynthesis of p‐coumaric acid derivatives, phytoalexin, and lignins that contribute to plant defense systems (Gill & Tuteja, 2010). Collectively, the levels of SOD, CAT, POD, APX, and PAL activities are the physiological characteristics to analyze and quantify the strawberry host resistance against pathogen infection. Our results proved that VOCs produced by *H. uvarum* could significantly induce the accumulation of defense‐related enzymes, such as SOD, POD, CAT, APX, PAL, and PPO. Importantly, the ability to improve contents of defense‐related enzymes is normally related to induction of system resistance in plants (Zhu & Ma, 2007), which suggests that *H. uvarum* VOCs could also be a potential microbe‐associated molecular patterns and function in the early perception status of the ISR of *H. uvarum*.

As an elicitor for improving the resistance of strawberry fruit by inducing the activities of antioxidant enzymes, our study also manifested that *H. uvarum* VOCs could reduce the contents of MDA in strawberry fruit throughout the storage period (Figure 4a), whereas the content of superoxide anion production rate was merely lower than nontreated strawberry fruit at the end of storage period (25 dpt, Figure 4b). MDA is the product of lipid peroxidation in plant cell membrane, as well as the generated rate of superoxide anion, which directly reacts with the degree of fruit ripening. Therefore, these two parameters partially represent the degree of damage to plant tissue (Macarisin, Droby, Bauchan, & Wisniewski, 2010). Thus, higher level of SOD, CAT, POD, APX, PAL, and APX activities and lower level of MDA content in strawberry fruit treated with *H. uvarum* VOCs indicate that VOCs of *H. uvarum* could trigger effective antioxidant defense system and probably delay senescence on strawberry fruit during postharvest storage.

Previous researches mostly focused on study of preventing fruit decay and fungal infections by microbe VOCs fumigation. Alström (2001) first proved VOCs produced by soil bacteria inhibit the growth of *Verticillium dahliae* effectively (Alström, 2001)*.* Chaurasia et al. (2005) indicated that VOCs of *Bacillus subtilis* have antagonistic effect on the pathogenic fungi and cause six pathogenic fungus spore and mycelium deformity (Chaurasia et al., 2005). Study of Arrarte, Garmendia, Rossini, Wisniewski, and Vero (2017) indicated VOCs produced by *Antarctic strains of Candidasake* control postharvest *Penicillium* and gray mold of apple (Arrarte et al., 2017). Huang et al. (2011) reported that VOC produced by *Candida intermedia* strain C410 suppressed the conidial germination and mycelial growth of *B. cinerea* and effectively controlled gray mold disease on strawberry (Huang et al., 2011). Moreover, they investigated the specific species of *C. intermedia* VOCs, such as esters, alcohols, alkenes, alkanes, alkynes, organic acids, ketones, and aldehydes, in which 1,3,5,7‐cyclooctatetraene and 3‐methyl‐1‐butanol were the most abundant. In another study, a total of 28 species were detected in *H. uvarum* VOCs, in which ethyl acetate and 1,3,5,7‐cyclooctatetraene were the most abundant (Qin et al., 2017). Indeed, there is a gap in our knowledge if microbe VOCs had positive effects on fruit quality during storage period.

Collectively, our study showed the VOCs produced by *H. uvarum* could improve the efficiency of scavenging active oxygen, slow down the process of strawberry peroxide, and delay the fruit senescence during the cold storage period. In addition, unlike traditional BCAs treatment, VOCs treatment on strawberry fruit does not require direct contact with fruit surface, which benefits them act as bio‐fumigants. We confirmed the functions of *H. uvarum* VOCs for regulating defense‐related enzymes and substances of reactive oxygen metabolism in strawberry in strawberry fruit. We also found that the *H. uvarum* VOCs could increase the contents of specific esters, such as methyl caproate, methyl octanoate, and methyl caprylate, in strawberry volatile emissions during postharvest storage. In our future study, it would be particularly interesting to understand the gene‐level regulation of *H. uvarum* VOCs on strawberry fruit and identify predominant functional ingredient in *H. uvarum* VOCs.

## 5 CONCLUSIONS

The present study showed that treatment with VOCs produced by the antagonist *H. uvarum* increased specific esters in strawberry volatile emissions during postharvest storage. Furthermore, the study indicated that treatment with VOCs can enhance the activity of defense‐related enzymes and inhibit the accumulation of MDA content and the production rate of superoxide anion. These results also showed that VOCs maintain the strawberry quality via inducing the expression of defense‐related enzyme genes. The molecular mechanism of VOCs treatment to increase strawberry resistance has not been elucidated yet. The future work should pay attention to investigate which signal pathway was induced on strawberry fruit treated with *H. uvarum* VOCs.

## CONFLICT OF INTEREST

It should be understood that none of the authors have any financial or scientific conflict of interest with regard to the research described in this manuscript.

## ETHICAL APPROVAL

This study does not involve any human or animal testing.
